# Generic Paradoxical Tensions, Appraisals, Work Motivation, and Performance: Insights From a Weekly Repeated-Measures Study

**DOI:** 10.3389/fpsyg.2021.700150

**Published:** 2021-08-19

**Authors:** Neha Tripathi

**Affiliations:** Department of Human Resources Management, Indian Institute of Management, Ahmedabad, India

**Keywords:** paradoxical tensions, cognitive appraisal, work motivation, performance, multilevel model

## Abstract

Drawing on the paradox theory, the author developed a theoretical model of appraisal–motivational responses to generic paradoxical tensions. The author postulated that paradoxical tensions are appraised both as a challenge and as a threat, in turn prompting mixed effects, positive and negative, on performance. The dual effects of paradoxical tensions are explained by the intermittent role of motivation toward work and a dispositional boundary condition—individual' adaptability—cross-situation variability of behaviors. The results from an eight-wave weekly repeated measures study spanning a period of 2 months (*N* = 178, total observations = 1,355) provided support for the proposed theoretical model. By illuminating the nuanced intraindividual psychological process, the present study brings forward novel insights on cognitive appraisals and motivations of paradoxical tensions advancing microfoundation of the paradox research.

Daily work life is imbued with paradoxes, the contradictory yet interrelated elements that exist simultaneously and persist over time (Smith and Lewis, 2011, p. 382). People face paradoxes of long- vs. short-term focus, stability vs. change, control vs. flexibility, self- vs. other-focus, empowerment vs. requirement enforcement, novelty vs. usefulness, and learning vs. performance daily at the workplace (Miron-Spektor et al., 2011; Smith, 2014; Zhang et al., 2015; Smith et al., 2016; Zheng et al., 2018; Wenzel et al., 2019; Zhang and Han, 2019). Paradoxes, simultaneous and synergistic coexistence of contradictions, result in tensions, defined as “stress, anxiety, discomfort, or tightness in making choices and moving forward in organizational situations” (Putnam et al., 2016, p. 68). Scholars argue about generalizability of paradoxes across a broad range of occasions spurring generic paradoxical tensions (Knight and Paroutis, 2017; Miron-Spektor et al., 2017). Extant research shows mixed, somewhat inconsistent, evidence on how individuals recognize and respond to tensions. A stream of research shows that embracing interwoven opposites and dualities promotes mental health, thereby enhancing creativity (Gaim and Wåhlin, 2016; Schad et al., 2016). In contrast, others document the dark side of paradoxes highlighting defensive responses against paradoxical tensions, resulting in anxiety and counter productivity (Lewis, 2000; Ashforth and Reingen, 2014). Recent theoretical developments accentuate the dual nature of paradoxes, describing them as a double-edged sword, evoking both virtuous and deleterious outcomes (Shao et al., 2019).

Although the double-edged effects of paradoxical tensions are theoretically argued (Smith and Lewis, 2011; Waldman et al., 2019), rigorous empirical studies examining such claims using the intraindividual approach are almost non-existent. Instead, the paradox research has devoted much attention on identifying paradoxes and their outcomes utilizing the qualitative method (Putnam et al., 2016; Schad et al., 2016). Putnam et al. (2016, p. 66) eloquently put it, “even though ‘process' is a celebrated attribute of paradox research, it has not always had center stage in paradox studies.” The psychological process, appraisal, and motivational, leveraging paradoxes to promote or hinder performance (i.e., small wins or subjective progress toward attaining work-related goals, Weick, 1984; Motowidlo et al., 1990; Motowildo et al., 1997) is woefully understudied. From both theoretical and practical standpoints, it is of utmost importance to unpack the appraisal–motivational responses to paradoxical tensions to advance scholarly understanding on effective coping from such tensions.

The author developed a processual model illuminating appraisal–motivational responses to paradoxical tensions. Appraisals—challenge and threat—have a distinct impact on the motivation of individuals and subsequent performance (Lazarus and Folkman, 1984; Deci and Ryan, 2000). Yet, paradox research provides limited insights on cognitive appraisals of tensions and is rather advanced in a piece-meal fashion, examining either benefits or adversaries of tensions. While some scholars argue that individuals harnessed paradoxical tensions as an opportunity for growth and learning (Osono et al., 2008; Smith, 2014; Gaim and Wåhlin, 2016; Knight and Paroutis, 2017), others highlight that tensions posed a threat and led to potential failure (Smith and Berg, 1987; Duffy et al., 2002; Ashforth and Reingen, 2014). Reconciliation of these pieces of contradictory evidence becomes important to empirically test the thesis how paradoxical tensions relate to appraisals, work motivations, and thus to performance.

Traits and abilities of individuals foster acceptance for and synthesis of interwoven and interdependent contradictions (e.g., paradox mindset, integrative thinking; Zhang et al., 2015; Miron-Spektor et al., 2017). The dispositional attributes that enable people to function effectively with paradoxical tensions are important to manage such tensions. Advancing this line of research, the author theorizes that the disposition, individual' adaptability, cross-situation variability (CSV) of behaviors enables the ability to appraise paradoxical tensions as a challenge, and thus, the motivation to perform. By illustrating the influence of adaptability on appraisal–motivational responses to paradoxical tensions, the present research provides thoughtful insights on the traits that promote paradoxes to yield performance (see conceptual model, Figure 1).

**Figure 1:**
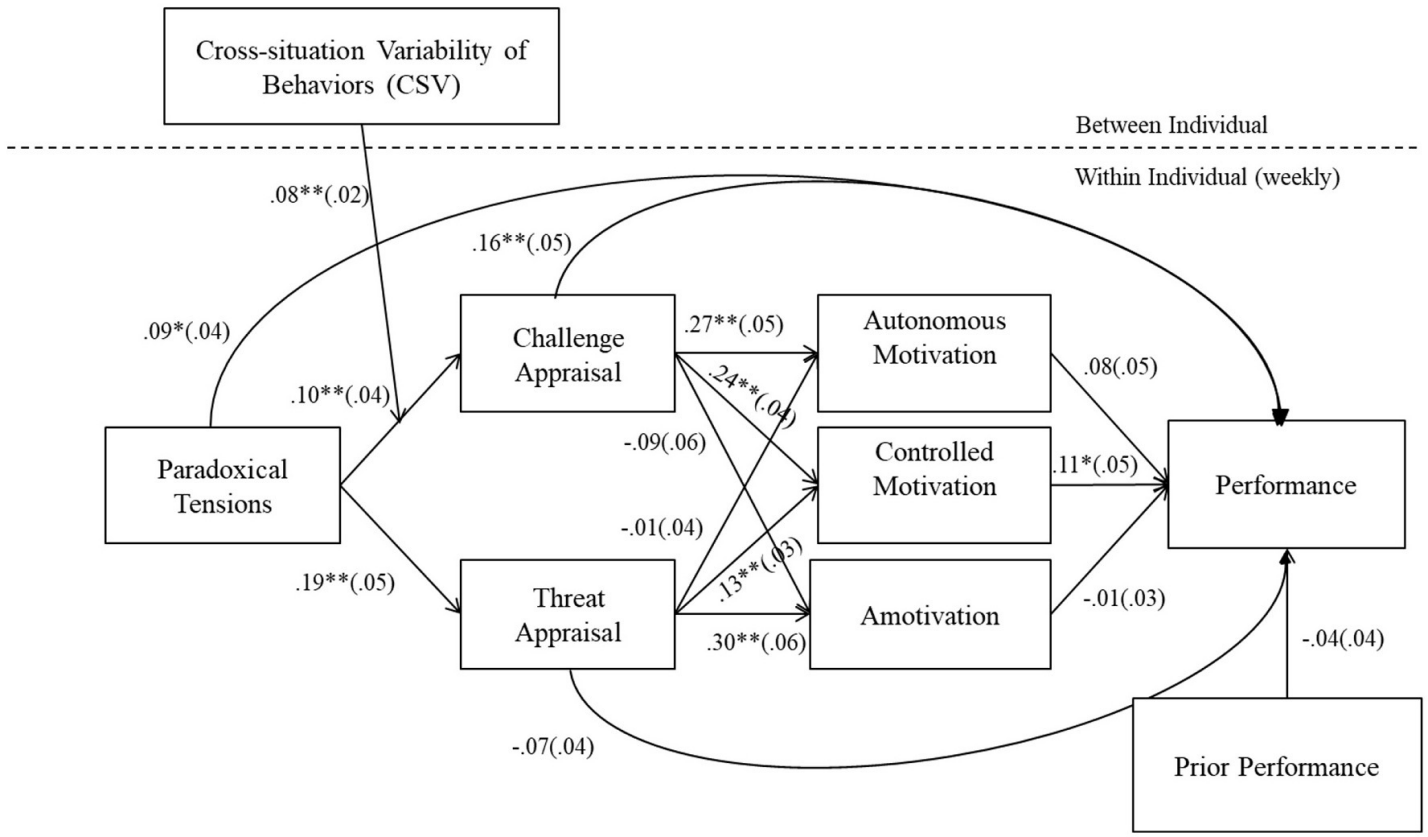
Effect of paradoxical tensions on weekly performance. *N* = 178, Number of observations = 1,355; Sample size is smaller for lagged and cross-level analysis; Unstandardized regression coefficients are reported. Between-person effects are controlled. *p < 0.05, **p < 0.01, Two-tailed test.

## Theory and Hypotheses

### Dual Nature of Paradoxical Tensions: Challenge and Threat Appraisals

Paradoxes are contradictory and interrelated elements that persist over time (Lewis, 2000; Smith and Lewis, 2011). Paradoxical tensions are experienced as stressful encounters often resulting from frustration, uncertainty, and inconsistencies that individuals face while dealing with contradictions (Smith and Berg, 1987; Vince and Broussine, 1996; Lewis, 2000; Smith and Lewis, 2011). Stressful experiences are relational in nature involving a transaction between the individual and the environment and are cognitively appraised as either challenging or threatening to the well-being of a person (Holroyd and Lazarus, 1982; Dewe, 1992). Cognitive appraisal unfolds in two steps, primary appraisal, a person judges what is at stake and, secondary appraisal, a person is concerned about controllability of the situation looking for coping strategies available to manage the demands (Lazarus and Launier, 1978; Lazarus and Folkman, 1984). A stress-inducing scenario is evaluated either as an opportunity for self-growth or as a threat of potential risk of failure.

Paradoxical tensions emanate from contradictory actions, ideas, and interests, and remain salient to the awareness of an individual (Shotter and Tsoukas, 2014). Paradox research highlights the dual nature of paradoxical tensions (Smith and Lewis, 2011; Waldman et al., 2019). Individuals may perceive contradictions and dualities as a challenge and embrace them as a source of motivation and as an inspiration for learning, creativity, and discovery. For instance, in a Danish Lego Company, Lüscher and Lewis (2008) observed that managers navigated paradoxical situations with initiatives and sense-making during organizational change, which enabled double loop learning and creative–integrative solutions to effectively manage organizational change. Likewise, others noted that the senior leaders juxtaposed innovation paradoxes by harnessing ambidexterity for exploration and exploitation, and by building integrative organizational architectures and processes (Smith and Tushman, 2005; Smith, 2014). However, scholars also highlighted an opposing side of paradoxical tensions that intensified cognitive paralysis (Smith and Berg, 1987; Lewis, 2000). Individuals formulated paradox by exclaiming feelings of “stuckness,” often described in paraphrases such as “I am not sure how to effectively delegate now,” “I am struck,” “damned if you do and damned if you don't” (Lüscher and Lewis, 2008; Putnam et al., 2016). Smith and Berg (1987) described paradoxes of group life perceived as “problems” and resolving these problems increased “stuckness” contributing to a vicious reinforcing cycle.

Vince and Broussine (1996) further illustrated when faced with paradoxical situations individuals experienced duality. In their study, managers pictorially depicted their experiences describing their encounters with paradoxes. The drawing of many managers reflected ambivalent cognitions and emotions such as “optimism and pessimism,” “hate and hope,” “challenge and doubt,” and “excitement and anxiety” (e.g., drawing an “angry” image of a politician along with a winning gold cup sailing toward a “benign” subset). Managers appraised organizational paradoxes with a sense of optimism and growth, and as a liberating process of removing barriers and overcoming adversity, at the same time with a sense of doubt, unfitness, and unrealizable idyll with a fear for personal catastrophe. These insights suggest that paradoxical tensions associate with both challenge and threat appraisals altogether.

*Hypothesis 1: Paradoxical tensions are positively related to the challenge and threat appraisals of an individual*.

### Mixed Effects of Paradoxical Tensions on Performance: Intermittent Role of Appraisals

The paradox research shows mixed pieces of evidence on the impact of paradoxes on performance. Paradoxes are found to be detrimental to work. For instance, paradoxes of high support and high undermining from the same source (i.e., superior or peers) showed a detrimental effect on Slovenian police officers lowering their self-efficacy and commitment (Duffy et al., 2002). Dualities and contradictions embedded within highly identified and highly disidentified promoted deviant behavior of higher tendency to organizational crime (Vadera and Pratt, 2013). Paradoxical tensions of idealism and pragmatism in a natural food cooperative ostensibly created dysfunctional teams (Ashforth and Reingen, 2014).

In contrast, paradoxes yielded performance and other work outcomes. Ingram and Roberts (2000) noted that hotel managers who had paradoxical friendship-relationships among competitors reported higher successful business and profit. Smith (2014) compared dynamic decision-making among top management teams in strategic business units and found that the teams that were engaged in the paradox of exploring and exploiting led innovation products more effectively as compared with teams that did not engage in the paradox of exploring and exploiting, and instead chose either exploring or exploiting. Pratt and Rosa (2003) reported that harvesting work–family paradoxes enhanced commitment from employees. Miron-Spektor et al. (2011) documented that paradoxical frames embedding contractions of novelty and usefulness enhanced creative performance among individuals. Paradoxes were thus found to promote innovation and performance (Andriopoulos et al., 2018).

Cognitive appraisals, challenge, and threat provide a plausible explanation to these pieces of contradictory evidence. When a person evaluates the situation as an opportunity for self-growth and identifies coping strategies available to manage the demands, stress is perceived as challenging. By contrast, when a person evaluates the situation as a source of failure and does not find appropriate coping strategies to manage the demands, stress is perceived a potential threat. These appraisals influence performance in different ways, such as challenge appraisal enhances performance, whereas threat appraisal adversely affects performance (Drach-Zahavy and Erez, 2002; González-Morales and Neves, 2015; Locke and Latham, 2019). Combined with Hypothesis 1, a dual-process model emerges whereby paradoxical tensions influence performance.

*Hypothesis 2: Paradoxical tensions have (a) positive indirect effect on performance via challenge appraisal and (b) negative indirect effect on performance via threat appraisals*.

### Intermittent Role of Work Motivation

Cognitive appraisals are linked to work motivation. Self-determination theory (Deci and Ryan, 2000) posits three broad categories of human motivation, namely, intrinsic, extrinsic, and amotivation. Intrinsic motivation refers to the drive of an individual for work solely for the pleasure of engaging in the work, whereas, extrinsic motivation refers to the drive of an individual for work for external reasons. Amotivation represents the lack of desire to engage at work. Based on regulatory styles extrinsic motivation is further divided into identified, introjected, and external regulation (Deci and Ryan, 2000). Studies show that intrinsic and identified regulation results in autonomous motivation, whereas introjected and external regulations result in controlled motivation (Fernet, 2011; Steingut et al., 2017; Slemp et al., 2020). Autonomous motivation refers to acting with volition, as when individuals engage in their work for inherent pleasure and satisfaction they experience (intrinsic motivation) and/or because they personally endorse the importance or value of their work (identified regulation). Controlled motivation refers to behaviors enacted under internal or external pressure, as when individuals perform their job to gain a sense of self-worth, or to avoid feelings of anxiety and guilt (introjected regulation), and/or because they are pressured by demands, threats, or rewards by an external agent (external regulation) (Gagné and Deci, 2005).

Paradoxical tensions prompt both challenge and threat appraisals (Hypothesis 1) giving rise to a sense of self-worth and favorable odds for learning as well as a sense of pessimism, hindrance, and blockage. In such situations, individuals synergize and find a manageable balance between opposing paradoxical elements, in this case, the opposing appraisals. For instance, Huy (2002) described that middle managers showed balance by simultaneously committing to change-oriented projects and, in contrast, also attending to employee adverse sentiments toward the change-oriented projects. Jarzabkowski et al. (2013) noted that managers actively responded to organizing, performing, and belonging paradoxes with an adjusting response cycle, which involved acceptance of both sides of paradox and recognition of their interdependence in achieving restructuring. These insights suggest that individuals manage paradoxical tensions by synergizing both internal urge and external pressure promoting controlled motivation to perform by gaining both a sense of self-worth as well as responding to pressure from competing demands.

*Hypothesis 3a: Paradoxical tensions are positively related to controlled motivation via opportunity and threat appraisals*.

The research on the effect of controlled motivation on work consequences shows pieces of mixed evidence, in that controlled motivation was found to associate with negative consequences (e.g., workaholism, burnout, and turnover intention; Richer et al., 2002; Fernet et al., 2008; Van den Broeck et al., 2011); did not associate with goal progress (Koestner et al., 2008); and was found to associate with the positive consequences (e.g., goal progress, Sobral, 2004; Gegenfurtner et al., 2009; Webber et al., 2010). To reconcile these pieces of contradictory evidence, Koestner et al. (2008, p. 1204) argued that the impact of controlled motivation is highly variable across situations such that “controlled motivation might facilitate goal progress, at least in the short term, in environments that provide frequent cues about the importance of striving for a particular goal.” This suggests that controlled motivation can enhance performance. Accordingly, the author posits,

*Hypothesis 3b: Challenge/threat appraisals and controlled motivation serially mediate the effect of paradoxical tensions on performance*.

### Moderating Role of CSV

Cross-situation variability refers to the ability of an individual to modify their behavior across situations (Snyder, 1974; Lennox and Wolfe, 1984). Adaptable individuals effectively tailor behavior to meet varying situational demands. In earlier works of Snyder (1974) on self-monitoring, CSV leveraged effective social participation, in that high self-monitors showed higher CSV to project a situationally appropriate façade, whereas low self-monitors reported unwillingness or lack of CSV to adapt behavior to press the situation (Snyder, 1974; Gangestad and Snyder, 2000). Individuals with adaptive abilities seem to perform particularly well in occupations that call for flexibility in dealings with diverse constituencies (Caldwell and O'Reilly, 1982). For instance, the research shows a positive association of the CSV of salespersons to their adaptive selling behavior (Spiro and Weitz, 1990). Sense making and adaptability of an Individual to adjust to different situations are further articulated as contributing factors in effectively managing paradoxes (Lewis, 2000; Smith and Lewis, 2011). Smith (2014) argued that the leaders are engaged in consistently inconsistent decision-making practices to deal with innovation paradoxes. These insights suggest that the adaptive ability of individuals harnesses paradoxes as a challenge appreciating the co-existence of contradictory perspectives (Smith and Tushman, 2005).

*Hypothesis 4: CSV of individuals strengthens the positive effect of paradoxical tensions on challenge appraisal*.

## Methods

### Participants and Procedures

A weekly repeated measures study was conducted with undergraduate students enrolled in the department subject pool in a reputed university situated in South-East Asia. The study was approved by the [University] Institutional Review Board (IRB: A-16-105). One hundred and eighty participants registered for the study in exchange for one credit. The participants first received a one-time baseline survey followed by weekly surveys for eight consecutive weeks (i.e., for 2 months). All the surveys were administered online. The participants reported their paradoxical tensions, appraisals, motivations, and performance every week. Since the experiences of a student at school could be affected by course work, and the teaching style of teachers, students were asked to report their experiences pertaining to a certain course, in which all the students were enrolled. All data came from a single source (i.e., students). After deleting incomplete and repetitive responses, baseline survey included 171 (out of 180 registrations) valid responses (response rate = 95%). Out of 171 valid responses, 46% of the participants were male. The majority of participants were Chinese (91%), remaining included Indian (6%), Malay (1%), Caucasian (1%), and others. The ages of the participants ranged from 19 to 25 years (mean = 20.7 years, SD = 1.33). Out of 180 registered participants, 178 provided 1,355 usable weekly observations (response rate = 94%, average cluster size = 7.61).

### Measures

All the measures used time reference of “during this week” with respect to the course all the participants were enrolled. Generic paradoxical tensions were measured using seven items developed by Miron-Spektor et al. (2017) on a five-point scale (“not at all,” “a little,” “somewhat,” “much,” and “very much”) (“I held ideas in mind that seem contradictory when appearing together,” “I have competing demands that need to be addressed at the same time,” α = 0.94). Ratings for all other measures were obtained on a six-point Likert scale in line with the research using Asian participants who show higher tendency to select “mid-point” (Wang et al., 2008). The psychometric properties (e.g., means, standard deviations, item–item correlations, item–total correlations, Cronbach's alpha, or factor loadings) of 4-, 5- 6-, 7-, 10-, and 11-point Likert scales are comparable and are not affected much by the number of points in the scale (Dawes, 2008; Leung, 2011). Challenge and threat appraisals were measured using six items developed by Drach-Zahavy and Erez (2002) (“my work seemed like a challenge to me,” α = 0.84; “my work seemed like a threat to me,” α = 0.90). Autonomous (four items), controlled (four items), and amotivation (two items) items were adapted from the work role motivation scale (Fernet, 2011) derived from SDT (Deci and Ryan, 2000) (“…for the pleasure that i from performing the class activities/tasks,” α = 0.84; “…because my position as student requires it,” α =.72; “I don't know. Most of the time, i am not really keen on performing such class activities/tasks,” α = 0.89). The participants reported their performance by responding to four items from Williams and Anderson (1991). Example item included “I fulfilled the responsibilities in the class” (α = 0.97). CSV was measured in the baseline survey using seven items from the concern for appropriateness scale developed by Lennox and Wolfe (1984), which is a widely used scale and is found to associate with adaptive behaviors (Spiro and Weitz, 1990) (α = 0.88, “…different situations can make me behave like very different people”).

#### Controls

To alleviate time-related confounds, weeks were controlled in the regression model. Performance of the previous week was controlled when predicting the performance of the concurrent week in the analysis to reduce any carry-over effects (Gabriel et al., 2019). Lastly, the research shows that relationship quality could influence the performance of an individual (Van den Broeck et al., 2016). Thus, indicators of relationship quality (interaction/satisfaction with instructor) were included as control variables.

### Analytical Strategy

Due to the nested nature of data (i.e., weekly observations nested within persons), multilevel path analyses (Preacher et al., 2010) using Mplus (Muthén and Muthén, 2017) was conducted. Estimates representing both within- and between-individual relationships were obtained in a single regression model. Variances and residual variances at the within and between levels are provided to infer effect sizes.

## Results

As shown in Table 1, a good proportion of within-person variance (>30%, see Klein and Kozlowski, 2000) occurred in paradoxical tensions (47%), opportunity appraisal (48%), threat appraisal (42%), motivations (35–44%), and performance (48%). Multilevel confirmatory factor analyses were conducted to establish construct validity (the results can be obtained from the author upon request). As depicted in Table 2 and Figure 1, paradoxical tensions enhanced both the challenges (γ_within_ = 0.10, *p* = 0.015; γ_between_ = 0.25, *p* = 0.02) and threat (γ_within_ = 0.19, *p* < 0.00; γ_*between*_ = 0.64, *p* < 0.00) appraisals. Challenge appraisal enhanced performance (γ_within_ = 0.16, *p* = 0.001; γ_between_ = 0.71, *p* < 0.00), whereas threat appraisal reduced performance (γ_within_ = −0.07, *p* = 0.079; γ_between_ = −0.33, *p* < 0.00). The indirect effect of paradoxical tensions on performance *via* challenge appraisal was positive [γ = 0.19, 95% CI (0.077, 0.308)] and *via* threat appraisal was negative [γ = −0.23, 95% CI (−0.333, −0.118)]. Both challenge (γ_within_ = 0.24, *p* < 0.00; γ_between_ = 0.65, *p* < 0.00) and threat (γ_within_ = 0.13, *p* < 0.00; γ_between_ = 0.15, *p* = 0.003) appraisals were positively related to controlled motivation. The indirect effect of paradoxical tensions on controlled motivation *via* challenge and threat appraisals was positive [γ = 0.11, 95% *CI* (0.039, 0.182)]. Controlled motivation was positively related to performance (γ_within_ = 0.11, *p* = 0.035; γ_between_ = 0.25, *p* = 0.023). The indirect effect of paradoxical tensions on performance *via* challenge and threat appraisals and controlled motivation was positive [γ = 0.07, 95% *CI* (0.005, 0.132)], supporting hypotheses 1–3.

**Table 1:** Means, SDs, ICCs, and correlations of the variables.

	**Variables**	**Mean**	**SD between**	**SD within**	**ICC1**	**1**	**2**	**3**	**4**	**5**	**6**	**7**	**8**	**9**	**10**
	**Level 1 (within)**														
1	Week	5.00	–	2.58	–	–	0.22^**^	−0.02	−0.08	0.45^**^	−0.00	0.02	0.17^**^	−0.09	0.34^**^
2	Interaction with instructor	1.67	0.63	0.57	0.59	0.08^**^	(0.88)	0.31^**^	0.39^**^	0.43^**^	−0.04	0.49^**^	0.21^**^	−0.18^**^	0.38^**^
3	Satisfaction with instructor	3.67	0.80	0.52	0.73	0.05	0.08^**^	(0.95)	0.05	0.59^**^	−0.19^**^	0.60^**^	0.47^**^	−0.53^**^	0.56^**^
4	Paradoxical tensions	2.25	0.93	0.65	0.53	0.05	0.17^**^	0.02	(0.94)	0.39^**^	0.47^**^	0.38^**^	0.22^**^	0.15^**^	0.16^**^
5	Challenge appraisal	4.24	0.77	0.51	0.52	0.08^**^	0.06^*^	0.23^**^	0.13^**^	(0.84)	−0.01	0.79^**^	0.67^**^	−0.41^**^	0.74^**^
6	Threat appraisal	3.10	1.03	0.67	0.58	0.05	−0.01	0.03	0.18^**^	0.35^**^	(0.90)	−0.02	0.16^**^	0.54^**^	−0.35^**^
7	Autonomous motivation	3.98	0.98	0.57	0.65	0.10^**^	0.13^**^	0.19^**^	0.10^**^	0.29^**^	0.09^**^	(0.84)	0.60^**^	−0.54^**^	0.59^**^
8	Controlled motivation	4.40	0.81	0.52	0.56	0.05	0.05	0.12^**^	0.13^**^	0.32^**^	0.26^**^	0.45^**^	(0.72)	−0.19^**^	0.53^**^
9	Amotivation	3.31	1.32	0.79	0.63	0.02	−0.08^**^	−0.08^**^	0.10^**^	0.03	0.25^**^	−0.03	0.19^**^	(0.89)	−0.49^**^
10	Performance	4.45	0.94	0.60	0.58	0.14^**^	0.05	0.24^**^	0.12^**^	0.22^**^	0.03	0.20^**^	0.19^**^	−0.01	(0.97)
	**Level 2 (between)**														
11	Cross-situation variability in behaviors (CSV)	4.24	0.89	–	–	–	0.03	−0.07	0.14	−0.11	0.22^**^	−0.07	−0.04	0.21^**^	−0.11

*N = 178, observations = 1,355, Correlations below diagonal represent within-person associations. Correlations above diagonal represent between-person associations. Correlations among level 1 and level 2 variables are calculated by aggregating level 1 variable across weeks (N = 171). Reliabilities are reported in parenthesis*.

* *p < 0.05*,

** *p < 0.01*,

*Two-tailed test*.

**Table 2:** Multilevel path analysis.

	**Dependent variable**											
	**Challenge appraisal**		**Threat appraisal**		**Autonomous motivation**		**Controlled motivation**		**Amotivation**		**Performance**	
**Variables**	**γ**	**S.E**.	**γ**	**S.E**.	**γ**	**S.E**.	**γ**	**S.E**.	**γ**	**S.E**.	**γ**	**S.E**.
**Within-person level**
Constant	2.03^**^	0.27	20.57^**^	0.34	−0.97^*^	0.40	0.80^*^	0.36	4.61^**^	0.56	1.25^*^	0.50
**Control variables**
Week	0.01	0.01	0.02	0.01	0.02	0.01	0.00	0.01	0.01	0.01	0.03	0.01
Interaction with instructor	0.02	0.03	−0.05	0.04	0.09^**^	0.03	0.02	0.03	−0.12^**^	0.04	−0.02	0.03
Satisfaction with instructor	0.23^**^	0.05	0.04	0.06	0.13^**^	0.04	0.06	0.04	−0.10^*^	0.05	0.23^**^	0.06
Prior Performance											−0.04	0.04
**Predictor variables**
Paradoxical Tensions	0.10^*^	0.04	0.19^**^	0.05	0.05	0.04	0.05	0.03	0.09	0.05	0.09^*^	0.04
Challenge Appraisal					0.27^**^	0.05	0.24^**^	0.04	−0.09	0.06	0.16^**^	0.05
Threat Appraisal					−0.01	0.04	0.13^**^	0.03	0.30^**^	0.06	−0.07	0.04
Autonomous Motivation											0.08	0.05
Controlled Motivation											0.11^*^	0.05
Amotivation											−0.01	0.03
**Between-person Level**
Interaction with instructor	0.13^*^	0.05	−0.26^*^	0.12	0.21^**^	0.07	−0.09	0.07	0.07	0.11	0.08	0.06
Satisfaction with instructor	0.37^**^	0.06	−0.15^*^	0.07	0.19^**^	0.06	0.15^*^	0.07	−0.40^**^	0.11	0.08	0.07
Paradoxical Tensions	0.25^**^	0.06	0.64^**^	0.10	0.88^**^	0.10	0.65^**^	0.10	−0.46^**^	0.16	0.09	0.07
Challenge Appraisal					0.03	0.06	0.15^**^	0.05	0.64^**^	0.08	0.71^**^	0.15
Threat Appraisal											−0.33^**^	0.07
Autonomous Motivation											−0.13	0.11
Controlled Motivation											0.25^*^	0.11
Amotivation											−0.03	0.07
Variance (within/between)	0.26/0.33		0.43/0.64		0.30/0.68		0.27/0.39		0.62/1.11		0.37/0.49	
Residual Variance (within/between)	0.24/0.17		0.42/0.45		0.27/0.22		0.23/0.20		0.57/0.53		0.32/0.14	
Δ*R*^2^	0.02/0.16		0.01/0.09		0.03/0.46		0.04/0.19		0.05/0.58		0.05/0.35	

*N = 178, number of observations = 1,355; sample size is smaller for lagged analyses; unstandardized regression coefficients are reported. Regression coefficients remain robust without including control variables*.

* *p < 0.05*,

** *p < 0.01, Two-tailed test*.

*The effect of paradoxical tensions on weekly performance*.

**Figure 2:**
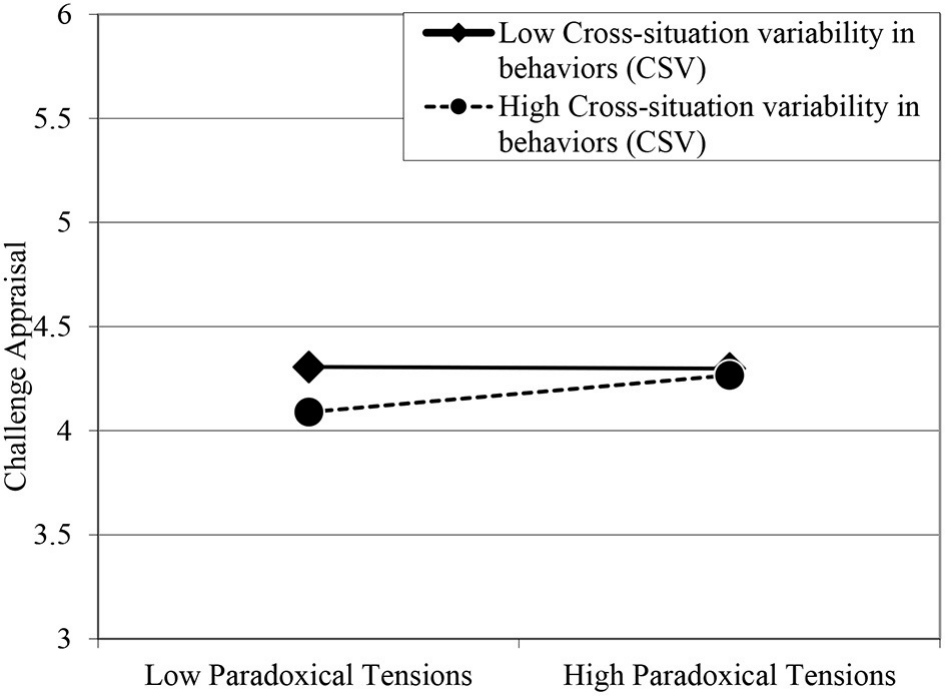
Moderating effect of cross-situation variability in behaviors (CSV) on the relationship between paradoxical tensions and challenge appraisal.

Multilevel moderation [level 1 model: Y = P0 + P1 (paradoxical tensions) + E; level 2 models: P0 = B00 + B01(CSV) + R0; P1 = B10 + B11 (CSV)] analysis was conducted to test Hypothesis 4 (B00 = 4.25, se = 0.04, *p* = 0.00; B01 = −0.07, se = 0.05, *p* = 0.16; B10 = 0.06, se = 0.02, *p* = 0.006; B11 = 0.08, se = 0.02, *p* = 0.002). Between-level predictor variable was grand-mean centered to alleviate any confounding effects relating to multicollinearity (Hofmann and Gavin, 1998). The moderating effect of CSV on the relationship between paradoxical tensions and challenge appraisal was positive and significant (γ_*slope*_ = 0.08, *p* = 0.002). Simple slope analysis was conducted at low and high (1 SD ± Mean) levels of moderator variable (low: intercept = 4.30, slope = −0.01; high: intercept = 4.17, slope = 0.14). As depicted in Figure 2, the positive relationship between paradoxical tensions and challenge appraisal was stronger when adaptability was high, as compared to when it was low, supporting Hypothesis 4.

## Discussion

The results showed that paradoxical tensions were appraised both as a challenge and as a threat prompting controlled motivation, in turn enhancing performance. CSV of individuals strengthened the link between paradoxical tensions and challenge appraisal. Overall, the results revealed the psychological appraisal-motivational mechanism whereby paradoxical tensions enhanced performance both at the within- and between-individual levels.

### Theoretical and Practical Implications

The present study significantly contributes to the emergent body of research on microfoundation of paradox (Miron-Spektor et al., 2017). First, paradox research, by large, has developed at the macrolevel identifying organizational paradoxes (see review, Schad et al., 2016). The present study is the first study to unravel performance consequences of everyday generic paradoxical tensions within/between individuals altogether. Second, the paradox research has predominantly utilized qualitative methods to identify paradoxical tensions. Such an approach is often critiqued to be in the “eyes of observer” lacking empirical evidence collected from the beholders to ensure the existence of paradoxes in real (Putnam et al., 2016). The present study provides empirical evidence from the beholders of how paradoxical tensions relate to cognitive appraisals. Third, the study illuminates novel explanatory mechanisms revealing the association of paradoxes to contradictory appraisals, involving both a sense of challenge and threat, prompting controlled motivation and performance, thus, enhancing scholarly understanding of the nuanced psychological factors that leverage paradoxical tensions to yield performance.

The present study offers practical implications to managers and organizations. Within personality research, leaders are sought to have “strong and stable personality” to effectively resist diverse situations (Duckworth and Quinn, 2009; Dalal et al., 2015). In contrast, the present study unravels the bright side of diversity or variability in behaviors articulating that adaptability of behaviors across different situations promote challenge appraisal, thus effectively coping from paradoxical tensions. Variability in the behavior of an individual can be conceived as a “fox-like” agile personality (and not the hedgehogs of organizations) to effectively manage contradictions and paradoxes (Silver, 2012). Smith et al. (2016, p. 67) further emphasized that, “…consistency is a vice. [One] must be able to appreciate multiple, often conflicting truths.” One must appreciate the inconsistencies, contradictions, and chaos leveraging on adaptability to effectively manage paradoxes. Thus, the managers are recommended to recognize the benefits of cross-situation variability in behaviors, navigating paradoxical tensions by acknowledging and appreciating contradictions and dualities as a challenge, in turn stimulating performance (Gibbons and Rupp, 2009).

## Strengths, Limitations, and Future Research Directions

Paradox research provides enriching evidence using cross-sectional, longitudinal, and quantitative methods (e.g., Denison et al., 1995; Smith, 2014; Zhang et al., 2015). Repeated measures methodology is useful to reduce the bias and error inherent in “in general” retrospective reporting. Despite being rigorous in the methodology, the present study is not without limitations. First, all the variables were self-reported, which may raise concerns about common method bias (i.e., inflation of relationships among study variables). However, repeated measures of these variables and multilevel analysis tested intra/interindividual effects simultaneously, mitigating the potential problems of response bias (Ilies et al., 2006). Second, the study utilized a sample of students instead of working professionals. Shen et al. (2011) documented that 40% of the samples published in the Journal of Applied Psychology from 1995 to 2008 used student sample and argued that “for research aimed at identifying general principles (i.e., can a phenomenon occur), student samples can be appropriate” (p. 1060). The present study examines the general principles pertaining to paradoxical tensions. Nonetheless, the author recommends future research to validate the findings in a work setting. Third, all the variables measured the experiences of the current week, thus, inference about causality was limited. Future research should replicate the results with more rigorous methods by separating study variables across time or by conducting experiments to establish causality. Lastly, the study was conducted with Asians, who are holistic thinkers (Peng and Nisbett, 1999), whereas the Western population, founded in Aristotle's either/or logic, excel in analytical cognition; thus they may adapt to paradoxes differently (Choi and Nisbett, 2000). Future research should validate the results in the Western population to establish generalizability.

## Data Availability Statement

The raw data supporting the conclusions of this article will be made available by the author, without undue reservation.

## Ethics Statement

The studies involving human participants were reviewed and approved by IRB National University of Singapore. The patients/participants provided their written informed consent to participate in this study.

## Author Contributions

The author confirms being the sole contributor of this work and has approved it for publication.

## Conflict of Interest

The author declares that the research was conducted in the absence of any commercial or financial relationships that could be construed as a potential conflict of interest.

## Publisher's Note

All claims expressed in this article are solely those of the author and do not necessarily represent those of their affiliated organizations, or those of the publisher, the editors and the reviewers. Any product that may be evaluated in this article, or claim that may be made by its manufacturer, is not guaranteed or endorsed by the publisher.
